# Postpartum behaviour as predictor of weight change from before pregnancy to one year postpartum

**DOI:** 10.1186/1471-2458-11-165

**Published:** 2011-03-16

**Authors:** Ellen Althuizen, Mireille NM van Poppel, Jeanne H de Vries, Jacob C Seidell, Willem van Mechelen

**Affiliations:** 1 van der Boechorststraat 7, 1081 BT Amsterdam, The Netherlands; 2 Department of Human Nutrition, Wageningen University, Wageningen, The Netherlands; 3 De Boelelaan 1085, 1081 HV Amsterdam, The Netherlands

**Keywords:** body weight change, postpartum, behavior

## Abstract

**Background:**

Postpartum weight retention affects many women and increases the risk of becoming overweight. The research objective was to study modifiable factors contributing to weight change at one year postpartum.

**Methods:**

In this prospective cohort, postpartum behavior, such as physical activity, sedentary behavior, sleep, and intake of total energy, total fat and saturated fatty acids of 118 Dutch women were assessed in 2003/2004 by self-report at 6 weeks, 6 and 12 months postpartum. Mean postpartum scores were computed for the behavioral measures. In linear regression models it was determined which factors were associated with average weight change from before pregnancy to one year postpartum. Furthermore, factors associated with substantial postpartum weight retention (≥ 5 kg) were also studied in logistic regression models.

**Results:**

At one year postpartum, the average weight of participants had increased by 0.9 kg (SD 4.4). Moreover, 20% of the women retained ≥ 5 kg. Women who perceived themselves more physically active than others were almost ten times less likely to retain ≥ 5 kg than women who perceived themselves equally active (OR = 0.11, 95%CI: 0.02 - 0.66). Exceeding the guideline for saturated fatty acid intake (OR = 3.40, 95%CI: 1.04 - 11.11), total gestational weight gain (OR = 1.14/kg, 95%CI: 1.01 - 1.27), and not having completed post high school education (OR = 5.13, 95%CI: 1.66 - 15.90) increased the odds of retaining ≥ 5 kg.

**Conclusions:**

Since one in five women had substantial weight retention postpartum, effective interventions for the prevention of weight retention are much needed. Future studies should evaluate whether interventions focusing on the identified modifiable postpartum factors are successful in reducing weight retention after childbirth.

## Background

The period after giving birth represents an important window for overweight prevention[1-3]. From conception to one year after giving birth, women are reported to gain between 0.5 and 3.0 kg on average [4-7]. Twelve to 25% of women cope with a substantial weight retention of 5 kg or more postpartum [4,7]. Weight retained after giving birth appears to be deposited in body fat centrally rather than peripherally [7,8]. Since overweight and a less favorable distribution of body fat are known risk factors for morbidity and mortality, it is important to study weight retention after women have given birth.

Gestational weight gain is the most consistently reported predictor of postpartum weight retention [5-7,9,10]. Other identified risk factors include non-white race, low socioeconomic status, and primiparity [11-13]. Only three studies reported on a multivariable approach including a variety of postpartum behavioral factors. Olson et al concluded that women who exercised "often" and those who perceived their energy intake to be decreased six to12 months postpartum had lower odds of retaining substantial weight [14]. Oken and colleagues concluded that hours of television viewing and trans fat intake increased the risk of substantial weight retention, whereas walking was a significant preventative factor [15]. Gunderson concluded that women sleeping ≤5 hours per day at six months postpartum had a threefold higher risk of substantial weight retention at one year postpartum [16].

Altogether, multivariable studies indicate that postpartum behaviour is of importance with regard to postpartum weight change. Such studies are very limited, however, and the need remains to improve our insight, being critical for the design of future intervention programmes [17]. Therefore, in addition to gestational weight gain and demographic variables, a number of postpartum behaviours such as physical activity, sedentary behaviour, sleep, and dietary intake were considered in the present paper, supplemented with data on meeting physical activity and dietary intake guidelines. These factors were chosen based on the previously mentioned studies, and from studies examining predictors of weight gain in general. The objective of the present study was to specify the relative importance of these repeatedly assessed postpartum behaviours with regard to both absolute and substantial (at least five kilograms) weight change at one year postpartum. The cut off point of five kilograms was chosen as a measure for identifying women who experience significant weight shifts after pregnancy [16,18].

## Methods

### Study design

This paper focuses on the longitudinal analysis of data reported at 30 weeks pregnancy and 6 weeks, 6 and 12 months postpartum. The data collection was conducted via mailed questionnaires from June 2003 to November 2004. Written informed consent was obtained from every respondent. The Medical Ethics Committee of the VU (Vrije Universiteit) University Medical Center, Amsterdam, the Netherlands approved the study protocol.

### Study sample

The Municipal Health Services of Amsterdam, which has a registry of almost all pregnant women in Amsterdam and the surrounding area, because they are responsible for blood screening in pregnancy, cooperated in the recruitment phase. First, 550 women of eighteen years or more were randomly selected by the Municipal Health Services to be invited to participate in our study. No upper age limit was used. We were allowed to send one mailed invitation, accompanied with some demographic questions, and one reminder invitation. There was no telephone contact during the recruitment phase for privacy reasons. Written informed consent was obtained from every respondent. After having given consent, reminder phone calls were made for follow-up questionnaires.

### Outcome measures

Two outcome measures were defined in order to study weight change 12 months after giving birth:

(1) total postpartum weight change (kg) as a continuous variable. This outcome measure was defined as the difference between postpartum weight (reported at 12 months postpartum) and prepregnancy weight (reported at 30 weeks pregnancy);

(2) substantial postpartum weight retention was defined as a weight retention ≥ 5 kg at 12 months postpartum, as a dichotomous variable (y/n).

### Behavioral covariates

Covariates were repeatedly assessed at 6 weeks, 6 and 12 months postpartum and mean postpartum scores of these measures were computed.

Physical activity (PA) was assessed by means of the SQUASH (the Short QUestionnaire to Assess Health-enhancing physical activity) a reliable and reasonably valid questionnaire, that can be used to rank adults according to their PA levels [19]. Minutes per day and number of days per week spent on activities were assessed in four domains: commuting, work, household work, and leisure time activities. Initially, the mean number of minutes spent per day in the first year postpartum on light PA (<4 Metabolic Equivalents, METs) and on moderate or vigorous PA (≥4 METs) were derived from the questionnaire. Second whether or not participants met the Dutch PA guideline [20], i.e. accumulating at least 30 minutes of moderate or vigorous physical activity on at least five days per week, was determined. The PA guideline was not deemed applicable six weeks after childbirth, since women are often recommended not resume their normal PA program before that time. The mean postpartum score for meeting the PA guideline was categorized as never/sometimes/always meeting the guideline at the three moments of measurement.

We also determined social comparison for PA to gain insight into how respondents perceived their individual PA level, comparing themselves with other women in the first year postpartum. At each measurement, women responded to the statement: "I think I am a lot less/a little less/equally/a little more/a lot more physically active than other women". The outer categories on both ends were combined with their neighboring category because they were hardly used. The resulting three categories (a little or a lot less/equally/a little or a lot more) were used in the analyses.

Time spent sleeping and time spent sitting and resting (lying down but not sleeping) were assessed. The following questions were used: "In the last week, how many hours did you sit/rest/sleep during a 24-hour day?" This was requested for week and weekend days separately, to reduce reporting bias owing to divergent activities on week and weekend days that are usual for working people. Finally, mean values over the three postpartum time points were computed for total hours per week spent on these behaviors.

The Dutch food frequency questionnaire was developed at the Division of Human Nutrition of the Wageningen University and validated to assess the intake of total energy, total fat and different types of fatty acids [21,22]. The original FFQ has been updated twice based on data of Dutch national food consumption surveys in 1992 and 1998 [23,24]. It is a 104-item questionnaire in which the women reported their food consumption of the previous four weeks. Frequency of foods used, as well as preparation methods, portion sizes and additions can be filled out. Using the Dutch food composition table of 2001 [25], the following measures were derived from this questionnaire: (1) total energy intake (kJ/day), (2) percentage of total energy intake derived from fat intake, and (3) percentage of total energy intake derived from saturated fatty acid intake. Furthermore, we determined whether or not participants met the guidelines of the Dutch Health Council for total fat intake (<40% of total energy intake) and for saturated fatty acid intake (<10% of total energy intake) [26]. Subsequently, mean scores of the three postpartum measurements were computed, illustrating whether participants always, sometimes or never met the guideline. Because the majority (75%) of participants always met the fat intake guideline, the "never" group (3%) was combined with the "sometimes" group (22%) in the analyses. With regard to saturated fatty acid intake, 37% never met the guideline, and the "sometimes" group (53%) was combined with the "always" group (9%).

Finally, in each questionnaire participants were asked to report on their breastfeeding practices. This variable was categorized into the following tertiles: never/up to four months/longer than four months. The cut point of four months postpartum was chosen since this time point is the time many women stop breastfeeding in the Netherlands.

### Demographics and other covariates

Total gestational weight gain was calculated by subtracting prepregnancy weight (reported at 30 weeks gestation) from end of pregnancy weight (reported at 6 weeks postpartum). Ethnicity was derived from the participant's parents' country of birth. An individual was considered to be European (which is in most cases Caucasian) when both parents were born in Europe. Further, educational level was operationalised as the highest level of education an individual reported to have completed. In this study, educational level was dichotomized into whether or not women had completed post high school education. Finally, participants were asked to report on their employment status (y/n), smoking status (y/n), family status (single or living alone/married or living together), and their health (excellent/very good/good/moderate/bad) [27].

### Statistical analyses

The analyses were conducted with SPSS 12.0.2. Dummy variables of categorical variables were generated and included in the regression models. Univariate predictors were selected (p < 0.2) for both outcomes separately. Two multiple regression models were developed by means of the stepwise forward method, with a significance level of p < 0.05: (1) a linear model with the dependent measure total postpartum weight change, and (2) a logistic model with the dependent measure substantial postpartum weight retention of 5 kg or more. All analyses were adjusted for prepregnancy body mass index (BMI). Finally, as a measure of model fit, the adjusted R-square was determined for the linear model.

## Results

**Figure 1:**
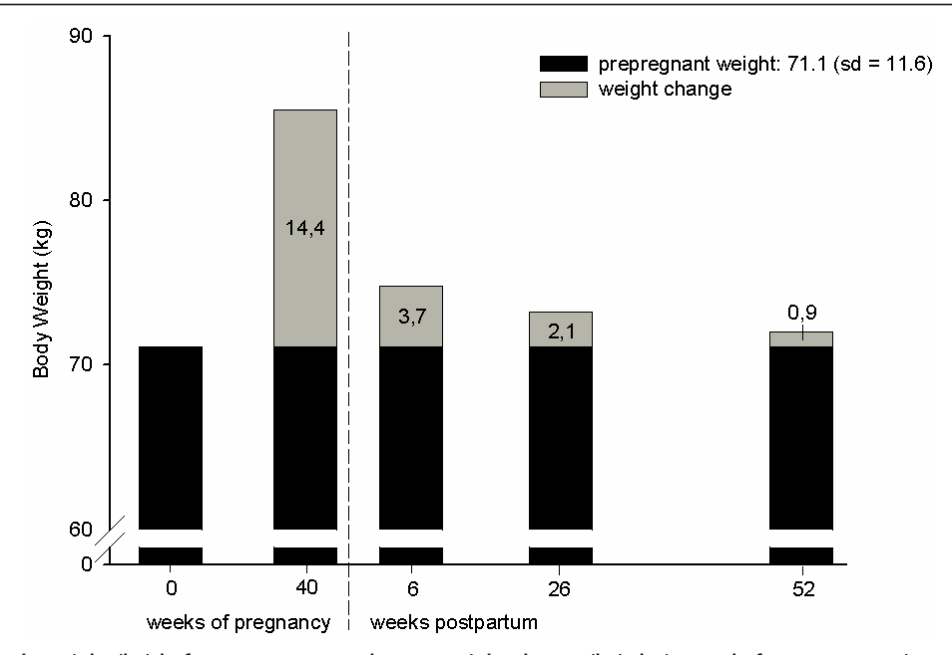
Mean body weight (kg) before pregnancy, and mean weight change (kg) during and after pregnancy (n = 118).

Initially, 168/550 (31%) women returned the first questionnaire. Subsequently, exclusions were made based on a preterm delivery at fewer than 36 weeks gestation (n = 3), when one or more questionnaires from 6 weeks, 6 or 12 months postpartum were not returned (n = 45), or when a subsequent pregnancy was reported within 12 months postpartum (n = 2), leaving a sample of 118 women for the analysis. Characteristics of our final study sample of 118 women are shown in Table 1. Most women (86%) were employed, and 97% were married or living with a partner. Almost half of the women (49%) were nulliparous, 40% primiparous, and 11% multiparous. Compared with the general population of Dutch pregnant women, our study sample had a comparable age and distribution of parity [28]. In our sample, 93% was European, which is considerably more than the 84% in the general population [28]. The women had a mean total gestation of 39.7 (SD 1.4) weeks. The last time they weighed themselves during pregnancy was at 39.2 (SD 1.5) weeks gestation on average. Mean total gestational weight gain was 14.4 (SD 4.9) kg (Figure 1), and ranged from 3.0 to 23.0 kg. In Table 2, data on self-reported lifestyle behaviour at 6 weeks, 6 and 12 months postpartum are presented. In this first year postpartum 10% of the women reported that they smoked.

**Table 1:** Population characteristics (N = 118)

	*mean (SD)*
Age, y	31.6 (4.3)
Age at menarche, y	13.2 (1.5)
Pre-pregnancy BMI, kg/m^2^	24.3 (3.7)

	***% (n)***

Pre-pregnancy BMI group	
*Underweight (BMI < 18.5)*	11 (8)
*Normal weight (BMI 18.5 - 24.9)*	52 (61)
*Overweight (BMI 25 - 29.9)*	30 (36)
*Obese (BMI *≥ *30)*	7 (8)
Educational level	
*Not completed post high school education*	32 (38)
*Completed post high school education*	68 (80)

**Table 2:** Self-reported lifestyle behaviour in the first year postpartum

Lifestyle behaviour	n = 118
Light physical activity, min/d, median (10-90^th^%)	323 (191-472)
Moderate and vigorous physical activity, min/d, median (10-90^th^%)	31 (10-93)
Participants meeting physical activity guideline, % (n)	
Never (not at 6 and 12 months pp)	40 (47)
Sometimes (only at 6 or 12 months pp)	33 (39)
Always (at 6 and 12 months pp)	27 (32)
Social comparison for physical activity, % (n)	
Less active than others	27 (32)
Equally active as others	36 (43)
More active than others	36 (43)
Sitting, hours/day, mean (SD)	6.5 (2.7)
Resting, hours/day, mean (SD)	1.2 (1.2)
Sleeping, hours/day, mean (SD)	7.8 (1.3)
Total energy intake, tertiles, % (n)	
Below average (< 628 kJ/day)	33 (39)
Average (628 to 803 kJ/day)	34 (40)
Above average (> 803 kJ/day)	33 (39)
Fat intake as percentage of total energy intake, mean (SD	32.6 (5.0)
Participants always meeting fat intake guideline, % (n)	75 (98)
Saturated fatty acid intake as percentage of total energy intake, mean (SD)	11.5 (2.6)
Participants always/sometimes meeting saturated fatty acid guideline, % (n)	63 (74)
Breastfeeding, % (n)	
Never	26 (31)
Up to 4 months	42 (49)
Longer than 4 months	32 (38)

Scores at 6 weeks, 6 and 12 months postpartum were computed into mean postpartum values.

### Absolute postpartum weight change

Postpartum weight change decreased from 3.7 kg (SD 4.2) at 6 weeks postpartum, to 2.1 kg (SD 4.3) at 6 months postpartum, to 0.9 kg (SD 4.4) at 12 months postpartum (Figure 1). Absolute weight change at 12 months postpartum varied between -10.0 and +11.0 kg, and was normally distributed.

In Table 3, the results of linear regression models are presented with absolute weight change at 12 months postpartum as the dependent variable. The final model (p < 0.01) explained 13% of the variance in absolute weight change. Women who perceived themselves to be more physically active than other women had significantly less weight retention. Also, women with a below average total energy intake had less weight retention compared with women with an average total energy intake. Sleeping showed an inverse association with absolute weight change, meaning that women who slept more retained less weight. Total gestational weight gain was significantly associated with absolute weight change.

**Table 3:** Multivariable correlates of average weight change and substantial (≥ 5 kg) weight retention at one year pp (n = 118)

*Absolute weight change (kg)*	B	95% CI
Social comparison for physical activity		
equally active (ref)	0	
less active	0.57	-1.35 - 2.49
more active	-2.43**	-4.53 - -0.71
Sleep (h/day)	-0.53*	-1.08 - -0.02
Energy intake ^b^		
average (ref)	0	
below average	-2.62**	-3.73 - -0.16
above average	-1.47	-3.35 - 0.42
Total gestational weight gain (kg)	0.18*	0.01 - 0.34

***Substantial weight retention (≥ 5 kg)***	**OR**	**95% CI**

Social comparison for physical activity		
equally active	1	
less active	1.90	0.59 - 6.08
more active	0.11*	0.02 - 0.66
Meeting saturated fatty acid intake guideline		
Yes	1	
No, exceeding guideline	3.40*	1.04 - 11.11
Total gestational weight gain (kg)	1.14*	1.01 - 1.27
Education		
Completed post high school education	1	
Not completed post high school education	5.13**	1.66 - 15.90

All models were adjusted for prepregnancy BMI.

* for p < 0.05, and ** for p < 0.01

### Substantial postpartum weight retention

In this study sample, 20% (23/118) of the women retained a substantial amount of weight of ≥ 5 kg at 12 months postpartum. The results of the final logistic regression model are shown in Table 3. Women who perceived themselves to be more physically active than others had an almost ten times lower odds of retaining a substantial amount of weight. Women who always exceeded the guideline of saturated fatty acid intake had a more than threefold higher odds compared with others. In addition, higher gestational weight gain increased the odds of retaining ≥ 5 kg. Finally, women who had not completed post high school education were more than five times as likely to retain substantial weight compared with women who did complete post high school education.

Meeting the guidelines for PA or fat intake did not significantly add to the final models of absolute or substantial postpartum weight retention. The same was true for the continuous measures of PA, for sitting, breastfeeding, and smoking behaviour, and for socio-demographic variables like ethnicity and parity. Prepregnancy BMI was not related to weight retention postpartum in either of these models.

## Discussion

Since studies that longitudinally looked at the influence of multiple postpartum behaviors on postpartum weight changes are scarce, we conducted a study in which the association of factors such as postpartum physical activity, sedentary behavior, sleep, and dietary intake with postpartum weight change were assessed. In our study, perceived physical activity, total energy intake, postpartum sleep, total gestational weight gain, and educational level were independently associated with weight change from before pregnancy to 12 months postpartum. These results indicate that total pregnancy weight gain combined with postpartum behaviours influence the degree of weight change following pregnancy.

A general comment should be made with regard to postpartum weight change. Studies on weigh retention generally reported weight changes of 0.5 to 3.0 kg [4-7,15,29,30], while they examined a period of more than one year. The total population, however, will also gain weight over the same time period [31]. Our participants retained on average 0.9 kg in about 18 months, which was similar to weight gain in a cohort of Dutch non-pregnant women of a comparable age, who gained 0.6 kg per year [31]. Of course these data originated from different samples, but they certainly illustrate that in a Dutch population weight change attributed to pregnancy may be less profound than in other populations.

Whatever the amount of weight gain caused by pregnancy, the fact remains that a substantial proportion of women are much heavier 12 months postpartum than they were before pregnancy. In our study 20% of women retained 5 kg or more. This is quite similar to other recent studies, conducted in the US or Europe, that found that between 13 and 20% gained 5 kg or more at 12 months postpartum [7,14,29,32].

### Behavioral predictors of postpartum weight change

Participants in the present study who perceived themselves to be more physically active than others were less likely to retain substantial weight after pregnancy, underlining conclusions of earlier studies which reported on beneficial associations between postpartum PA and body weight [15,30,33]. In contrast, the more in-depth measures derived from our activity questionnaire were not associated. There was only a moderate correlation between "social comparison for PA" and minutes spent in "at least moderate PA" derived from the SQUASH (r = 0.41, p = 0.00; data not shown), which indicates that the brief item is likely to reflect a different concept of PA behaviour.

In the present study, an association between total energy intake and weight retention was found. Previous studies also found that relatively high levels of energy intake were linked to increased (risk of substantial) postpartum weight change [14,15,33-35]. However, not only total energy intake may be important, also food quality (i.e. specific nutrients) may play a role in weight change, and advice about intake of specific nutrients might be more effective than advice about total energy intake [26,36]. Thus far, only Oken and colleagues investigated measures of food intake in the postpartum period [15]. They concluded that trans fat intake was a predictor of substantial postpartum weight gain. In addition to this finding, the present study shows that high intakes of saturated fatty acids in the first year postpartum are associated with a higher risk of substantial weight retention. Given the scarcity of studies, the relationship between postpartum weight retention and other qualitative measures of food intake, e.g. fibre intake, deserves further research.

Sedentary behaviour in the postpartum period was only studied before by Oken and colleagues, who demonstrated that women who watched fewer than two hours of television were about five times less likely to retain ≥ 5 kg [15]. In our study, evidence for a relationship with sitting or resting time was absent, perhaps because we did not ask for a specific behaviour such as television watching, but the total time of sitting per day, which is possibly more difficult to estimate.

Interestingly, a higher mean score of sleep in the first year postpartum was a significant protective factor for absolute weight change 12 months postpartum. Gunderson et al also found a protective effect of sleep for weight retention in the postpartum period [16]. It is hypothesised that women who sleep less because of long hours caring for their babies [37] may be especially susceptible to weight gain, or not able to lose weight postpartum. Further assessment of the relation between postpartum weight retention and (determinants of) sleep in the postpartum period is, however, warranted.

Breastfeeding status was no significant predictor in either of the presented models. Breastfeeding in relation to postpartum weight changes has been studied frequently, but the evidence is inconclusive; ranging from negative to weak positive associations, or not a significant association at all [14,37-39].

### Limitations and strengths

Several limitations should be taken into account when our results are interpreted. The low response rate is the main drawback of this study. The resulting relatively small sample size undermines the discriminating power of the presented data, which may partially account for the limited explained variance of the linear regression model (13%). In addition, the overrepresentation of European and well-educated participants undermines the generalisability of our findings. The foremost objective of the current study was to describe the strength and direction of significantly associated factors of postpartum weight change, which was not undermined by this selectivity. Another limitation was that self-reported measures were used, where objective, more precise measures would have been preferred. Furthermore, some of the instruments were not validated, and none were validated in pregnant or postpartum women. Body weight, energy intake and physical activity are especially known to be subject to reporting bias with increasing overweight [41,42]. It was not possible to confirm the data objectively owing to practical limitations (e.g. assessing body weight is not common practice in Dutch midwifery). Still, since both outcome variables were based on change scores, and assuming that underreporting of body weight is rather consistent over time, reporting bias might not have affected the outcome measures as such. Lastly, we combined data from all three time points postpartum into one score for average postpartum behaviour. This strategy did not allow looking at the importance of early or late postpartum behaviour for postpartum changes in weight.

The foremost strength of our study is its prospective design. Repeatedly assessed measures, which were computed into mean postpartum scores in the first year postpartum, decreased standard errors of the correlates that were studied. Besides this, finding comparable results in both regression models can be seen as confirmation that the reported predictors are indeed of value with regard to postpartum weight change. Last but not least, reporting on multivariate associations, hereby controlling for co-linearity (which is common between behaviours - especially with regard to PA and energy intake), enabled us to draw conclusions about the relative importance of the assessed measures.

## Conclusions

Postpartum lifestyle factors such as PA, sleep, energy intake, and meeting the guideline for saturated fatty acid intake, total gestational weight gain and educational level were identified as factors related to postpartum weight change. Future interventions should evaluate whether guidance with regard to the identified modifiable postpartum factors may be able to reduce weight retention after childbirth, hereby improving maternal health.

## Competing interests

The authors declare that they have no competing interests.

## Authors' contributions

MvP initiated and was PI of the study. MvP and EA designed the study. EA performed the

statistical analysis assisted by MvP and JdV. EA wrote the manuscript. JdV, JS and WvM participated in the design of the study and contributed to the manuscript. All authors read and approved the final manuscript.

## Pre-publication history

The pre-publication history for this paper can be accessed here:

http://www.biomedcentral.com/1471-2458/11/165/prepub
